# Identification and characterization of duck plague virus glycoprotein C gene and gene product

**DOI:** 10.1186/1743-422X-7-349

**Published:** 2010-11-27

**Authors:** Bei Lian, Chao Xu, Anchun Cheng, Mingshu Wang, Dekang Zhu, Qihui Luo, Renyong Jia, Fengjun Bi, Zhengli Chen, Yi Zhou, Zexia Yang, Xiaoyue Chen

**Affiliations:** 1 Avian Diseases Research Center, College of Veterinary Medicine of Sichuan Agricultural University, Ya'an, Sichuan, 625014, China; 2 Key Laboratory of Animal Diseases and Human Health of Sichuan Province, Ya'an, Sichuan, 625014, China; 3 Epizootic Diseases Institute of Sichuan Agricultural University Ya'an, China

## Abstract

**Background:**

Viral envelope proteins have been proposed to play significant roles in the process of viral infection.

**Results:**

In this study, an envelope protein gene, gC (NCBI GenBank accession no. EU076811), was expressed and characterized from duck plague virus (DPV), a member of the family herpesviridae. The gene encodes a protein of 432 amino acids with a predicted molecular mass of 45 kDa. Sequence comparisons, multiple alignments and phylogenetic analysis showed that DPV gC has several features common to other identified herpesvirus gC, and was genetically close to the gallid herpervirus.

Antibodies raised in rabbits against the pET32a-gC recombinant protein expressed in Escherichia coli BL21 (DE3) recognized a 45-KDa DPV-specific protein from infected duck embryo fibroblast (DEF) cells. Transcriptional and expression analysis, using real-time fluorescent quantitative PCR (FQ-PCR) and Western blot detection, revealed that the transcripts encoding DPV gC and the protein itself appeared late during infection of DEF cells. Immunofluorescence localization further demonstrated that the gC protein exhibited substantial cytoplasm fluorescence in DPV-infected DEF cells.

**Conclusions:**

In this work, the DPV gC protein was successfully expressed in a prokaryotic expression system, and we presented the basic properties of the DPV gC product for the first time. These properties of the gC protein provided a prerequisite for further functional analysis of this gene.

## Background

Duck plague virus (DPV), or duck enteritis virus (DEV), is an important pathogen of ducks, which has caused serious losses in commercial duck production in domestic and wild waterfowl as a result of mortality, condemnations, and decreased egg production[1]. DPV is classified as the subfamily alphaherpesvirinae of the family herpesviridae based on the report of the Eighth International Committee on Taxonomy of Viruses (ICTV), but has not been grouped into any genus[2].

The genome of DPV is composed of a linear, double-stranded DNA, with 64.3% G+C content which is higher than any other reported avian herpesvirus in the subfamily alphaherpesvirinae[3]. To date, more and more DPV genes have been identified, such as UL24[4-6], UL31[7,8], UL35[9,10], UL51[11,12], dUTPase[13], and gE[14] gene. However, the key genes and their functions remain to be elucidated, especially the viral envelope protein genes. Viral envelope proteins are particularly important because of their role in the virus-host relationship, including recognition, attachment and penetration of the virus into susceptible cells. In 2006, the DPV genomic library was successfully constructed in our laboratory, and one envelope protein gene, gC (NCBI GenBank accession no. EU076811) was characterized[15-18], but the basic properties and biological functions of this envelope protein are not known.

gC is a major component of the virion envelope and is proved to be a multifunctional protein. gC homologues of herpes simplex virus type 1 (HSV-1), pseudorabies virus (PRV), and bovine herpesvirus type 1 (BHV-1), is the primary attachment protein, interacting with cell surface heparan sulfate proteoglycans (HSPG), thus mediating efficient virus attachment to the cells[19-24]. gC of HSV-1, varicella-zoster virus (VZV) and PRV[25-28] is also a major determinant for virulence. In case of Marek's disease virus (MDV), gC is required for horizontal transmission, together with US2, UL13 in combination[29]. Furthermore, gC has been demonstrated to be a critical immune evasion molecule, and the two glycoproteins, gC and gE, have a synergistic effect on mediating immune evasion[30,31]. And gC of HSV-1 and -2, BHV-1, PRV, and Equine herpes virus types 1 and 4 (EHV-1 and -4) has been reported to bind complement component C3[32-34], thus modulating complement activation. Although nonessential for virus infectivity of cultured cells, gC is a highly antigenic glycoprotein, of which the importance in eliciting immune responses has been well documented for many herpesviruses[35-39]. However, whether the product of DPV gC gene shares these functions remains to be determined.

To begin addressing questions regarding gC properties or functions, we cloned and expressed the gC gene from DPV in the prokaryotic expression system, raised antiserum that recognizes the gC protein and revealed its temporal transcription course and subcellular localization in DPV-infected DEF cells. This work might provide a foundation for further studies on the function of DPV gC.

## Results

### Cloning, prokaryotic expression and antigenicity analysis of the recombinant protein

**Figure 1:**
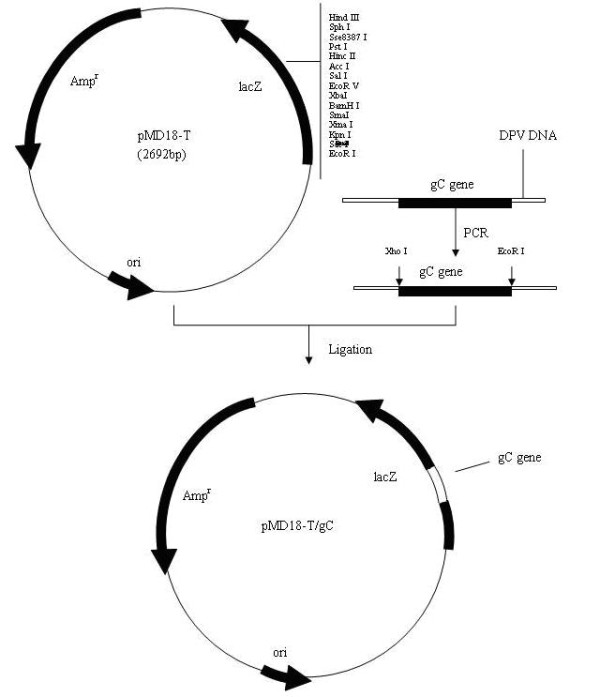
**Schematic diagram of gC gene cloned into the pMD18-T cloning vector**.

**Figure 2:**
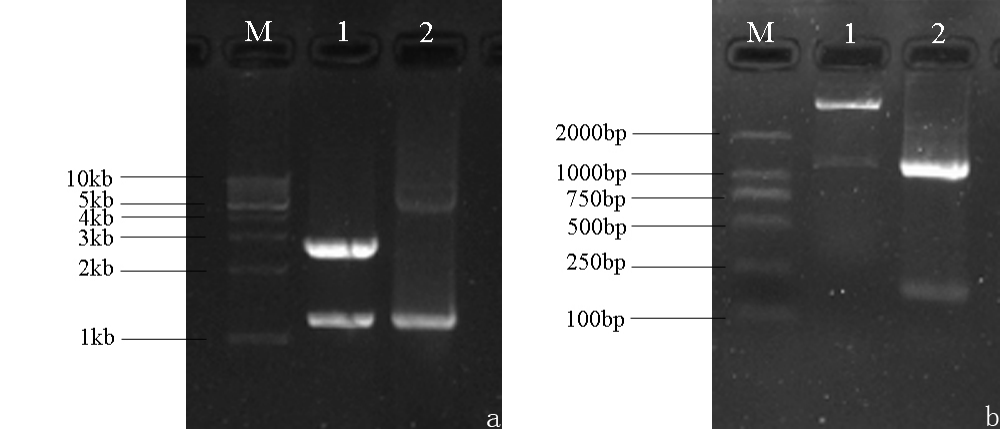
**Identification and characterization of pMD18-T/gC and pET32a-gC with restriction enzyme and PCR-based amplification.** (a) Identification of pMD18-T/gC with restriction enzyme and PCR-based amplification. Lanes: 1, pMD18-T/gC digested with EcoRI and XhoI; 2, product amplified from pMD18-T/gC. M, DNA marker. (b) Characterization of the recombinant plasmid pET32a-gC by restriction digestion and PCR-based amplification. Lanes: 1, pET32a-gC digested with EcoRI and XhoI; 2, product amplified from pET32a-gC. M, DNA marker, marker III.

**Figure 3:**
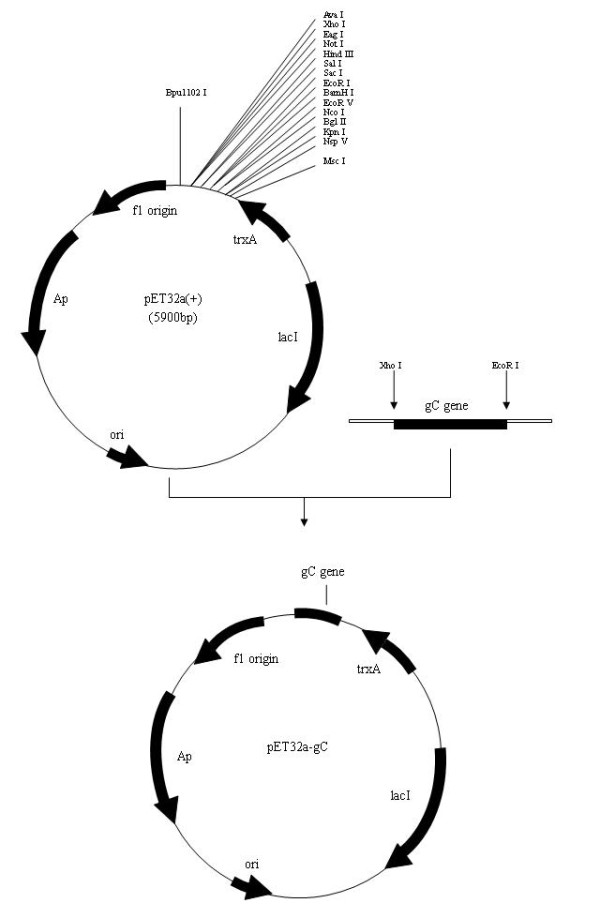
**Construction of the recombinant expression plasmid pET32a-gC**.

**Figure 4:**
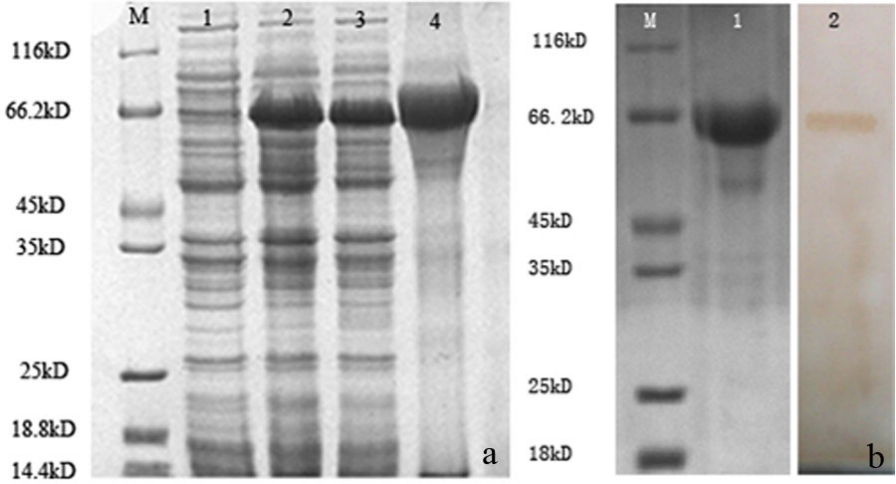
**Expression, purification and antigenicity analysis  of pET32a-gC recombinant fusion protein.** (a) Expression of pET32a-gC recombinant fusion protein. Lanes: 1, pET32a-gC, non-induced; 2, pET32a-gC, induced by 0.6 mmol/L IPTG; 3, pET32a-gC, induced by 1 mmol/L IPTG; 4, IPTG-induced inclusion body fraction. (b) Purification and antigenicity analysis of the recombinant fusion protein. Lanes: 1, Purification of the fusion protein by electric elution; 2, Western blot result of pET32a-gC recombinant fusion protein. M, Protein molecular mass markers.

DPV gC gene from the genomic DNA was amplified and cloned into a T/A cloning vector pMD18-T, generating a recombinant cloning plasmid pMD18-T/gC (Figure 1). The recombinant plasmid was confirmed by DNA sequencing, PCR and restriction digestion (Figure 2a). The gC gene fragment, which was obtained by digestion of pMD18-T/gC with EcoRI and XhoI, was ligated into the fusion expression vector pET32a(+) (Figure 3) and identified by PCR and restriction digestion (Figure 2b). After confirmation, a positive clone was submitted to DNA sequencing and the result confirmed that the gC gene was in frame with the N-terminal His6 tag within the pET32a(+) multiple cloning sites (data not shown). Then this recombinant plasmid, pET32a-gC, was transformed into Escherichia coli BL21 (DE3) which, following induction with IPTG, expressed large quantities of the pET32a-gC recombinant protein (Figure 4a), and this recombinant protein was purified by gel and electric elution (Figure 4b). In order to examine the reactivity and specificity of the recombinant fusion protein, Western blot analysis was carried out. As shown in Figure 4b, the anti-DPV serum specifically recognized a 65 kDa band, which corresponded to the theoretical molecular mass of pET32a-gC.

### Transcriptional analysis of DPV gC gene

**Figure 5:**
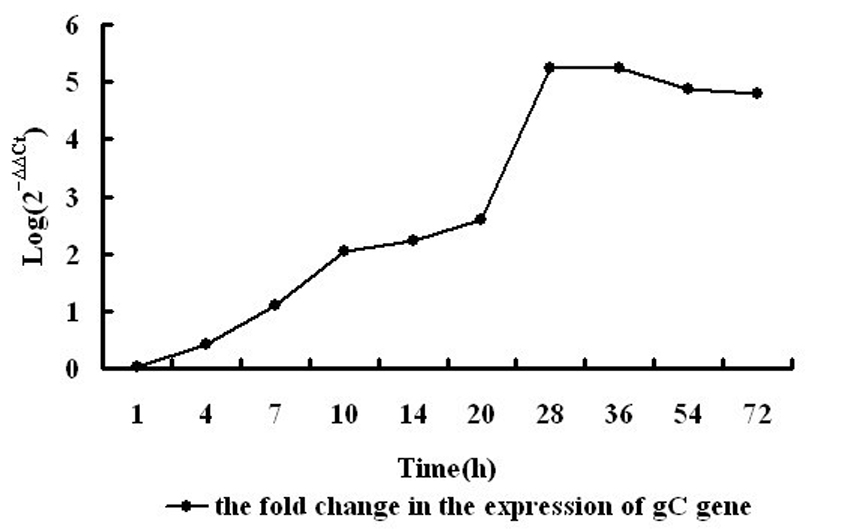
**Transcriptional analysis of DPV gC gene**. Total RNA was isolated from the cells at each time point and converted to cDNA. Samples of cDNA (1 μl) were amplified using real-time quantitative PCR and SYBR green detection. Presented is the fold change in the expression of gC gene.

FQ-PCRs were used to detect the expression of the DPV gC gene during viral infection. Total RNA was isolated from mock-or DPV-infected cells at indicated times, and then cDNA was synthesized using reverse transcriptase. Aliquots of cDNA at each time point were used as template for real-time PCR reactions containing primers either for gC gene or for β-actin. Table 1 presents data from experiments where the target (gC) and reference (β-actin) were amplified in separate wells. The 2^-ΔΔCt ^method was used to calculate relative changes in the gene expression determined from quantitative real-time PCR experiments. As shown in Figure 5 the level of gC mRNA had been increasing since 4 hpi, peaked between 28 and 36 hpi, and then declined.

**Table 1:** Sample spreadsheet of data analysis using the 2-ΔΔCt method

Time	gC		β-actin		ΔΔCt	**2**^**-ΔΔCt**^	Log
	**MeanCt,**_**TimeX**_	**MeanCt,**_**Time0**_	**MeanCt,**_**TimeX**_	**MeanCt,**_**Time0**_			**(2**^**-ΔΔCt**^**)**
1 h	21.3	24.5	10.6	13.7	-0.1	1.071773	0.03
4 h	20.7	24.5	11.3	13.7	-1.4	2.639015	0.42
7 h	19.6	24.5	12.5	13.7	-3.7	12.99603	1.11
10 h	16.5	24.5	12.5	13.7	-6.8	111.4304	2.05
14 h	14.1	24.5	10.7	13.7	-7.4	168.897	2.23
20 h	14.6	24.5	12.4	13.7	-8.6	388.0234	2.59
28 h	11.3	24.5	17.9	13.7	-17.4	172950.5	5.24
36 h	11.9	24.5	18.5	13.7	-17.4	172950.5	5.24
54 h	14.4	24.5	19.8	13.7	-16.2	75281.1	4.88
72 h	18.9	24.5	24	13.7	-15.9	61147.25	4.79

### Time course expression of DPV gC protein

**Figure 6:**
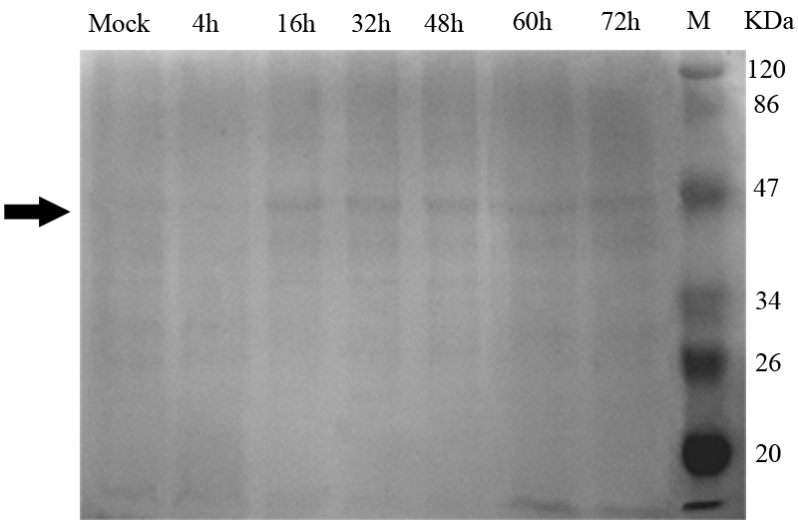
**Expression of gC protein in DPV infected cells**. Proteins isolated from mock- (lane 1) or DPV-infected cells at different times (lanes 2 to 7) were analyzed by Western blot analysis with gC antiserum. The arrow shows the expected position for DPV gC (about 45 kDa). The electrophoresis migration of molecular mass markers is shown on the right.

In this experiment, DEF cells were mock infected or infected with DPV, and at 4, 16, 32, 48, 60, 72 hpi, cell suspensions were harvested and lysed in RIPA buffer. Equal amounts of cell lysates were resolved by SDS-PAGE, and proteins on the gel were electrophoretically transferred to PVDF membrane and subjected to Western blot analysis with rabbit anti-DPV gC serum. The result, shown in Figure 6 revealed that a 45 kDa protein was readily detected as early as 4 hpi and seemed to be present at increased levels at 48 hpi.

### Subcellular localization

**Figure 7:**
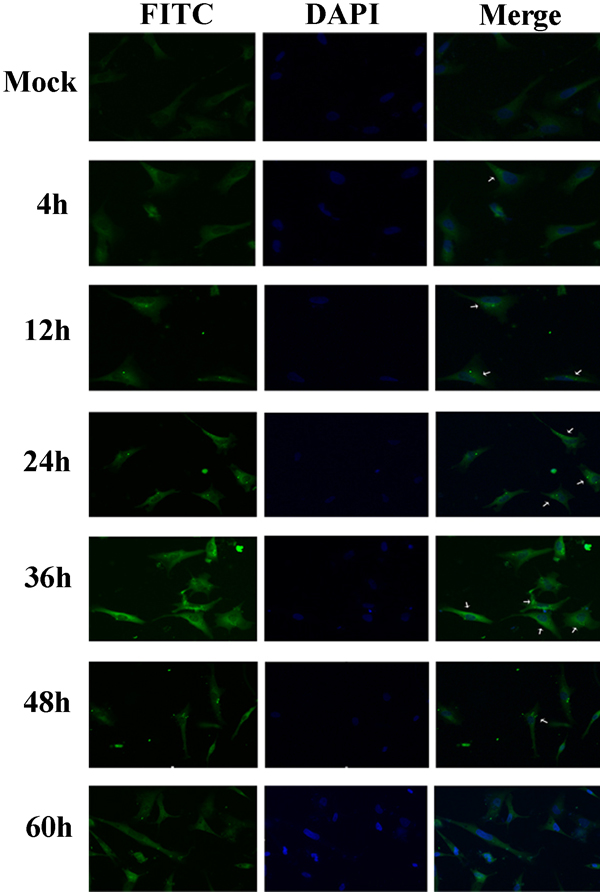
**Subcellular localization of DPV gC**. DEF cells were infected with DPV for 4, 12, 24, 36, 48 or 60 h and the cells were fixed, permeabilized and stained with anti-DPV gC serum and FITC-conjugated goat anti-rabbit antibody, followed by DAPI. The arrows indicate the DPV gC FITC fluorescence staining. Mock-infected cells were used as a negative control.

The subcellular localization of gC protein was examined by indirect immunofluorescence staining. At various times after infection, DEF cells infected with DPV were fixed with 4% paraformaldehyde, treated with 5% bovine serum albumin (BSA) to block nonspecific binding and reacted with the DPV gC antiserum. Specific fluorescence became detectable only in the cytoplasm of infected cells as early as 4 hpi. At later times of infection, the protein converged at the perinuclear region of the cytoplasm. And after 60 hr, fluorescence was gently dispersed (Figure 7).

## Discussion

In this study we expand our initial observations, in which we identified and characterized the DPV gC gene and found that it has homologues in some herpesviruses sequenced to date. Analysis of the predicted 432 amino acid DPV gC protein indicates that it has several features common to other herpesvirus glycoproteins. There is a hydrophobic sequence of about 22 amino acids at the amino terminus that corresponds to a signal peptide and a 23-amino-acid hydrophobic sequence near the carboxy terminus that is predicted to span the membrane of the virus envelope. Amino acid sequence comparison revealed that DPV gC gene displayed similarities of 29.9%, 29.5%, 31.7% to this gene from MeHV-1, MDV1, MDV2, respectively. Further detailed analysis showed that it possessed a Marek's disease glycoprotein A conserved structural domain between the residues 170 and 425, indicating that the DPV gC and its counterpart in MDV may have similar functions.

As a first step toward the study of the gC protein, rabbit polyclonal antiserum specific to this protein were raised using an Escherichia coli BL21-produced recombinant gC fusion protein as antigen. For this purpose the plasmid pET32a-gC was constructed. When expressed in Escherichia coli BL21, this plasmid expresses the gC gene fragment along with a His6 tag attached to the N terminus. High levels of the resulting 65 kDa fusion protein were expressed in Escherichia coli BL21 following induction by IPTG. The induced fusion protein was purified as described in Methods.

To examine the reactivity and specificity of DPV gC protein, Western blot experiments were performed. The result showed that the fusion pET32a-gC protein was recognized by the rabbit anti-DPV IgG, indicating that the protein had good immunogenicity. Then the fusion pET32a-gC protein was used as antigen to produce the rabbit polyclonal antiserum specific for gC. The fusion pET32a-gC protein was recognized with the pET32a-gC antiserum by Western blot and the antiserum specifically reacted with a protein of approximately 45 kDa protein in DPV-infected DEF cells. These results indicated that the antiserum had a high level of reactivity and specificity. Therefore, we used this polyclonal antiserum for further experiments to characterize the gC protein of DPV.

During a productive infection of cultured cells, genes of herpesvirus have been found to be expressed in a temporally regulated cascade, in which immediate-early (IE) genes are expressed first, followed by early (E) genes and finally by late (L) genes[40]. Late genes are subdivided into two categories as leaky-late (γ_1_) or strict-late (γ_2_). The γ_1 _genes can be suboptimally expressed in the absence of viral DNA synthesis, whereas the γ_2_, have a strict requirement for viral DNA synthesis. gC gene of many herpesviruses has been identified as a γ_2 _gene, which is highly dependent upon the IE protein ICP27 during viral infection[41-47]. In HeLa cells infected with HSV-1 (5 PFU/cell), the transcript for the γ_2 _gC was present from 2 to 8 hpi and the relative increase in gC transcript was detected by 8-h RNA hybridization[48]. Levine M[49] reported that gC protein was expressed at high levels within a single HSV replication cycle of about 10 to 14 h. From our data, the level of DPV gC mRNA had been increasing since 4 hpi, with maximal amounts between 28 and 36 hpi and the maximum gC expression was achieved by 48 hpi. These results demonstrated that the expression of this gene occurred at the late stage of infection, which was to some extent consistent with the results from the previous observations. In this report, DPV gC mRNA and protein peak levels were detected much later compared to HSV-1 gC gene, probably because of the difference in the cell type and the dose of infection.

Currently, little is known about the subcellular localization of the herpesviruses gC. To assemble clues to the function of the gene product, we investigated the subcellular localization of DPV gC in infected cells by indirect immunofluorescence experiments. The presented results showed that cytoplasm fluorescence first appeared in DPV-infected cells at 4 hpi. At later times of infection, the specific fluorescence was localized predominantly intracellularly in a perinuclear region which probably corresponds to the rough endoplasmic reticulum and/or Golgi apparatus of the infected cells, in which viral glycoproteins were synthesized and/or modificated.

## Conclusions

In this work, we characterized the gC gene of DPV, including the prokaryotic expression, antibody preparation, gene temporal transcription/translation course and subcellular localization. We found that the expression of this gene appeared at the late stage of viral infection and the gC protein showed a pronounced cytoplasmic staining in infected cells. These properties of the gC protein provide a foundation for further functional analysis of this gene.

## Methods

### Cells and virus

Duck embryo fibroblasts (DEF) were cultured at 37°C with 5% CO_2 _in minimal essential medium (MEM) containing 10% fetal bovine serum (FBS) (Hyclone, Logan, Utah, USA), 100 U/ml penicillin, and 100 μg/ml streptomycin.

DPV CH virulent strain was obtained from the Avian Disease Research Center of Sichuan Agricultural University. For infection, DPV of 2.2 × 10^7 ^TCID50/ml was employed.

After DPV inoculation, the DEF were incubated in MEM containing 3% FBS. Usually, the maximum virus titers could be obtained 72 h postinfection (hpi) when the cytopathic effect (CPE) was over 75%.

### PCR amplification and plasmid construction

A pair of primers (5'-CGGAATTCCAAAACGCCGCACAGATGAC-3' and 5'-CCCTCGAGGTATTCAAATAATATTGTCTGC-3') was designed and used to amplify DPV gC gene from the genomic DNA. The amplified PCR product was cloned into a T/A cloning vector pMD18-T (TaKaRa), generating a recombinant cloning plasmid pMD18-T/gC (Figure 1). After verified by PCR, restriction analysis and DNA sequencing (TaKaRa), the gC gene fragment, which was obtained by digestion of pMD18-T/gC with EcoRI and XhoI, was ligated into prokaryotic vector pET32a(+) (Novagen) (Figure 3), which was digested previously with the same restriction enzymes. The recombinant plasmid, named pET32a-gC, was confirmed by PCR, restriction enzyme digestion and DNA sequencing (TaKaRa).

### Prokaryotic expression, protein purification and antibody preparation

pET32a-gC was transformed into Escherichia coli BL21 (DE3) and the bacteria were induced for 4 h with 0.6 mM IPTG at 37°C to express the fusion protein. The fusion protein was purified from inclusion bodies by gel and electric elution. To test the antigenicity of the recombinant fusion protein, proteins separated by 12% SDS-PAGE were subsequently subjected to Western blot analysis with rabbit anti-DPV serum. The purified recombinant protein was then mixed with an equal volume of Freund's complete adjuvant (Sigma) and used to immunize rabbits by intradermal injection, followed by two additional intradermal inoculations with Freund's incomplete adjuvant once every 7 days and the last inoculation with the purified recombinant protein. After the fourth immunization, anti-DPV gC serum was collected. Then, the purified IgG polyclonal antibodies were obtained by purification using caprylic acid and ammonium sulfate precipitation and High-Q anion exchange chromatography.

### FQ-PCR

Total RNA was isolated from mock-or DPV-infected cells at different times (1, 4, 7, 10, 14, 20, 28, 36, 54, 72 hpi) and was reverse transcribed at 37°C for 120 min using random primer according to the manufacturer's instructions. The real-time PCR assays were performed using an iCycler iQ™ real-time PCR detection system (Bio-Rad Lab., Hercules, CA, USA). The primers used for PCR amplification were as follows: forward primer 5'-GAAGGACGGAATGGTGGAAG-3' and reverse primer 5'-AGCGGGTAACGAGATCTAATATTGA-3', which amplify a 78-base pair (bp) fragment of DPV gC gene, and for the endogenous control gene β-actin, forward primer 5'-CCGGGCATCGCTGACA-3' and reverse primer 5'-GGATTCATCATACTCCTGCTTGCT-3'. The amplification was performed in a 20 μl reaction mixture containing 9 μl of POWER High-Capacity cDNA Reverse Transcription Kits SYBR Green PCR master mix (Applied Biosystems), 0.5 μl of each primer, 1 μl of cDNA template and 9 μl of sterile ultra pure water. Three replicates of each reaction were performed. The PCR condition consisted of one cycle of 1 min at 95°C followed by 40 two-step cycles of 30 sec at 94°C and 30 sec at 60°C. Homogeneity of products from each reaction was confirmed by melt curve analysis. Analysis of the real-time PCR data was carried out using the comparative ΔΔCt method[50]. The fold change in expression of gC gene relative to the endogenous control gene (β-actin) at various time points was calculated as Fold change = Log(2^-ΔΔCt^), where ΔΔCt = (Ct, _Target_-Ct, _Reference_)_Time x_- (Ct, _Target_-Ct, _Reference_)_Time 0_.

### Western blot analysis

DEF were either mock infected or infected with DPV of 2.2 × 10^7 ^TCID50/ml, harvested at various indicated times (4, 16, 32, 48, 60, 72 hpi), lysed on ice for 30 min with an equal volume of radioimmunoprecipitation assay (RIPA) buffer (50 mM Tris-HCl, pH 7.4, 150 mM NaCl, 1% Triton X-100, 1% sodium deoxycholate, 0.1% SDS, and 1 mmol/l phenylmethylsulfonyl fluoride) and centrifugated at 13,000×rpm for 15 min at 4°C[13]. Then equivalent amounts of the cell lysates were electrophoresed on 12% SDS-PAGE followed by staining with Coomassie Brilliant Blue R-250 and simultaneously electrophoretically transferred to a polyvinylidene fluoride (PVDF) membrane (Bio-Rad Lab., Hercus, CA, USA) in a transfer buffer at 120 V for 90 min. For Western blot analysis, purified rabbit polyclonal antibodies IgG was used as the primary antibody at a dilution of 1:50, followed by horse peroxidase (HRP) conjugated goat anti-rabbit IgG at a dilution of 1:5000 (KPL Inc., Gaithersburg, Maryland, USA) as the secondary antibody.

### Subcellular localization

DEF cells, grown on coverslips in six-well plates, were mock infected or infected with DPV of 2.2 × 10^7 ^TCID50/ml and then fixed with 4% paraformaldehyde for 15 min at room temperature at different times (4, 12, 24, 36, 48, 60 hpi). After blocking in PBS containing 5% bovine serum albumin (BSA) at 37°C for 1 h, the cells were incubated with purified rabbit polyclonal antibodies IgG (1:100 dilution) specific for recombinant DPV gC at 4°C overnight, rinsed three times for 10 min each with PBS and incubated with fluorescein isothiocyanate (FITC)-conjugated goat anti-rabbit IgG (Sino-American Biotechnology Co., Shanghai, China) for 1 h at 37°C. 4,6-Diamidino-2-phenylindole (DAPI; Sigma) staining was used to visualize the cell nuclei. Fluorescent images were viewed and recorded with the Bio-Rad MRC 1024 imaging system.

## Competing interests

The authors declare that they have no competing interests.

## Authors' contributions

BL carried out most of the experiments and drafted the manuscript. CX participated in the previous studies and helped to draft the manuscript. AC and MW critically revised the experiment design and the manuscript. DZ, QL, RJ, FB, ZC, YZ, ZY and XC helped with the experiment. All authors read and approved the final manuscript.
